# Marine Microbial Exopolysaccharides (EPSs): Untapped Bio-Reserves

**DOI:** 10.3390/polym17243249

**Published:** 2025-12-06

**Authors:** Bilal Aslam, Muhammad Hassan Khalid, Sulaiman F. Aljasir

**Affiliations:** 1 Department of Veterinary Preventive Medicine, College of Veterinary Medicine, Qassim University, Buraydah 51452, Saudi Arabia; b.aslam@qu.edu.sa; 2 Institute of Microbiology, Faculty of Life Sciences, Government College University Faisalabad, Faisalabad 38000, Pakistan

**Keywords:** AMR, marine microorganisms, exopolysaccharides, industrial biotechnology, climate change

## Abstract

Antibiotic discovery occurs at a snail’s pace, coercing researchers to find novel and promising alternatives to tackle antimicrobial resistance (AMR). Marine microbial exopolysaccharides (EPSs) have emerged as one, considering the recent recognition of these substances as a significant bioactive compound. This manuscript is intended to relate the identified molecular features of marine-driven EPS and applications in the field of biomedical sciences. The current review pointed out the ecological merits of such polymers in agriculture sector. Biochemical structure and the controlling mechanisms of EPS production in marine microbes are considered key features as well. Climate-induced factors impacting the production, composition, and functionality of EPSs are scrutinized. Last but not least, it draws biological insights from medical, industrial, and biotechnology sectors, thereby highlighting their linkage between antimicrobial innovation, industrial biotechnology, and environmental sustainability, while also describing the concerns that need to be resolved, like translation of laboratory results into marketable products.

## 1. Introduction

As a rich source of novel biopolymers, the marine microbiome produces exopolysaccharides (EPSs) endowed with significant and targeted biological functions, while living in oligotrophic settings, an extreme environment with low temperature and high pressure. Such compounds are well-recognized due to their promising application in different industries and scientific fields, i.e., the food and agriculture industry, pharmaceutical and cosmetics applications, astro-biological applications, environmental bioremediation, etc., [1,2].

EPSs are high-molecular-weight polysaccharides composed of repeating monosaccharide units [3]. They are produced extracellularly and accumulate around the bacterial cell surface. They differ in their structure and composition because the kind or strain of the microbe, as well as the surroundings in which they are generated, define their character [4]. It is documented that several types of bacterial exopolysaccharides are quite potent antimicrobial compounds [5,6]. Marine microbial EPSs have demonstrated efficacy against numerous pathogens, such as *Pseudomonas aeruginosa*, *Vibrio* spp., *Escherichia coli*, and *Staphylococcus aureus* [7].

Currently, the focus is on identifying new marine microorganisms that produce EPS and exhibit distinguishable characteristics. Marine organisms have been utilizing their creative metabolisms and also developing their defense mechanisms to live in extremely hostile environments. This fact may enable them to produce these cutting-edge bioactive compounds when compared to the rest of the natural environments [8]. The activity of these microorganisms can be connected to various mechanisms, like altering the physicochemical characteristics of the surfaces involved, whether living or non-living, changing the expression of genes that are related to quorum sensing and adhesion, and eliminating biofilms by their natural biosurfactant properties [9].

**Figure 1:**
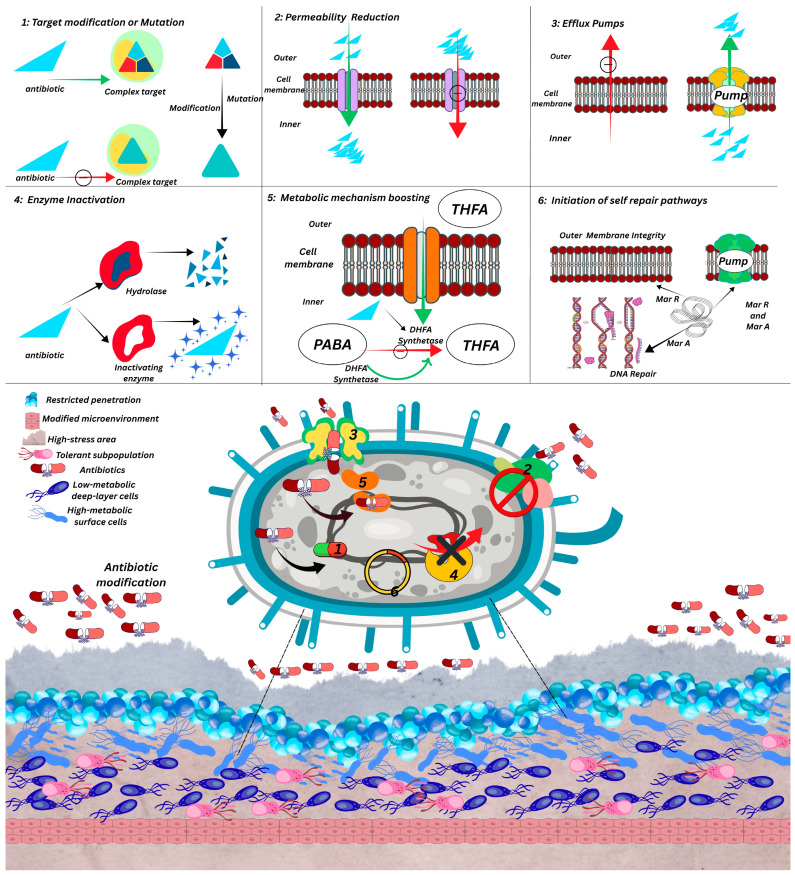
Schematic representation of the multifaceted mechanisms underlying bacterial resistance against antibiotics. (**1**) Target modification or mutation alters drug-binding sites, reducing antibiotic affinity. (**2**) Reduced permeability limits drug entry through changes in membrane porins. (**3**) Efflux pumps actively expel antibiotics, maintaining sub-lethal intracellular concentrations. (**4**) Enzymatic inactivation neutralizes antibiotic molecules via hydrolysis or chemical modification. (**5**) Metabolic pathway enhancement enables alternative synthesis routes, such as THFA production via DHFA synthetase. (**6**) Activation of repair pathways restores membrane integrity and DNA structure following antibiotic-induced damage. The lower panel depicts biofilm-associated protection, where restricted penetration, metabolic heterogeneity, and tolerant subpopulations further reinforce antibiotic resistance [14].

Though EPSs have been known for their wide applications, their maximum potential still needs to be exploited, e.g., fighting AMR, food safety, next-generation environment protection, etc., [10,11]. The incessant emergence of multidrug-resistant (MDR) strains, elevating crisis of AMR due to scarce novel antimicrobials, impels researchers to discover potential alternatives to combat this global health concern [12]. Owing to the potential application of such bio compounds, these can be used to tackle resistant strains and AMR crisis (Figure 1). Recent past investigations displayed that, due to the diverse marine microbial community, research interests have been extended to marine environment to find promising antimicrobial compounds [13].

Herein, the current review discusses the possible advantages that were not directly highlighted through the use of marine-derived exopolysaccharides (EPSs) along with their hidden activities, e.g., antibacterial, antibiofilm, etc. Moreover, it presents the merits of these compounds in different fields, like medical, drug delivery, and agriculture, simultaneously considering the impact of climate change on the production and the physiochemical properties of the marine microbial EPSs.

### Literature Search Strategy and Selection Criteria

In order to achieve a wide-ranging and impartial examination of the subject, a systematic literature search method was used in this review. The articles published in peer-reviewed journals were taken from Google Scholar, PubMed, Scopus, and Web of Science with a time span of 2010 to 2025, with the emphasis on the latest developments in the characterization, biological activities, and biotechnological applications of marine microbial exopolysaccharides (EPSs). Some of the search terms were the following: “marine EPS”, “marine exopolysaccharides”, “antibiofilm EPS”, “EPS antimicrobial activity”, “marine polysaccharides drug delivery”, “marine EPS agriculture”, and “EPS climate change regulation”.

The inclusion criteria were the following:

(i) Studies that described the isolation, biochemical characterization, regulatory pathways, or functional applications of EPS from marine microorganisms;
(ii) Microorganisms were to be marine bacteria, cyanobacteria, microalgae, and marine fungi only;
(iii) Full-length peer-reviewed publications;
(iv) Articles in English.

The exclusion criteria were the following:

(i) Studies about the EPS from terrestrial microbes;
(ii) Patents, conference abstracts, or papers without experimental details;
(iii) Studies on polysaccharides that are purely synthetic or chemically synthesized.

The first screening revealed more than 150 records. After duplicates were removed and irrelevant or low-quality studies were excluded, 92 articles were determined to be suitable for inclusion in this review. Also, additional papers were identified by cross-referencing to ensure that all relevant research findings were fully covered.

## 2. Biochemical Properties of Marine Exopolysaccharides

The rheological properties of EPSs produced by microorganisms are of a unique nature and can be demonstrated through the use of diverse characterization methods that take advantage of their feature of forming viscous, plastic-like fluids. In addition, EPSs play the role of reservoirs of energy and carbon [15]. Typically, they are composed of monosaccharides and non-carbohydrate substituents such as succinate, acetate, phosphate, and pyruvate [16]. Carbohydrates (glucose, galactose, mannose, rhamnose, fucose, etc.) are the main components of EPS, along with very small amounts of proteins, uronic acids, lipids, or nucleic acids [17]. Glucose- and galactose-rich EPSs are known to enhance biological activities, including anti-inflammatory effects, thereby increasing the overall bioactivity of these polymers. In addition, rare sugars such as rhamnose and fucose impart unique functional properties, making EPS valuable for applications in cosmetics, pharmaceuticals, medical devices, and functional foods [18].

**Figure 2:**
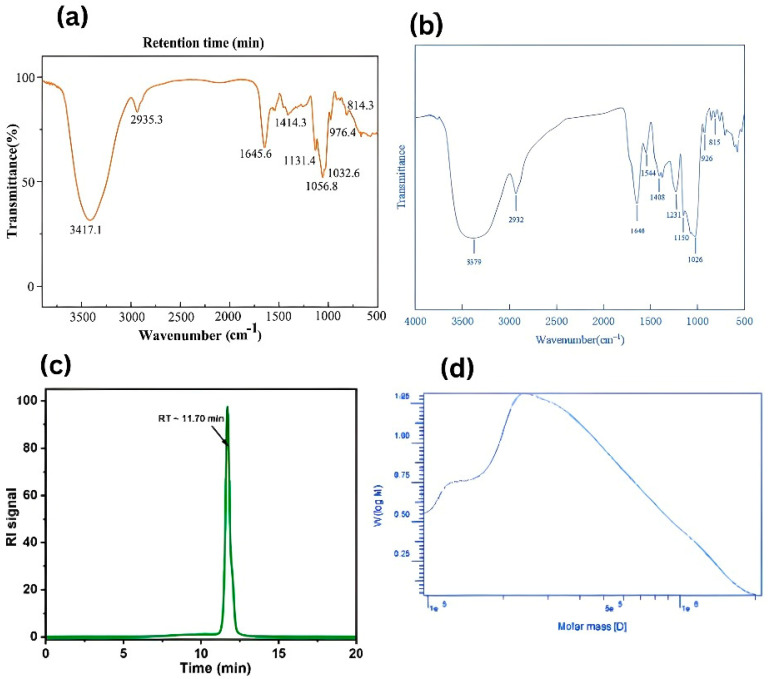
(**a**,**b**) FTIR spectra for functional groups of marine microbe *Pediococcus pentosaceus* EPS adapted and modified from previously published studies: [21], Frontiers in Microbiology, 13:923522, and [22] Microorganisms, 10(1):18. While (**c**) depicts the SEC profile of Marine EPS molecular weight, adopted and modified from [24] Journal of Fungi 10(9):659, and (**d**) depicts the HPGPC-based molecular-weight distribution of marine EPS, adopted and modified from [25] Journal of Applied Pharmaceutical Science 9(11):010–018.

The sulphuric acid-phenol technique revealed a 93.2% carbohydrate content of EPS synthesis in a marine bacterium, namely, *Pediococcus pentosaceus* [19]. *Pediococcus pentosaceus* EPS-2 fraction was chemically characterized as a hyperbranched glucomannan composed mainly of mannose and glucose. Its backbone included 1,2-α-Manp, 1,3-α-Manp, 1,2,6-α-Manp, 1,6-α-Glcp, and 1,4-α-Glcp, with side chains of T-α-Manp linked via α-1,2- and α-1,6-glycosidic bonds [20]. Functional groups in the EPSs can also come into play in relation to binding metal ions, antioxidant activity, as well as biofilm properties. *Pediococcus pentosaceus*, FT-IR peaks of EPS-E8 showed hydroxyl (3417 cm^−1^), alkane C–H (2935 cm^−1^), and glycosidic C–O–C/C–O–H bonds (1131–1032 cm^−1^), confirming its polysaccharide structure with α-glycosidic linkages (976, 814 cm^−1^) [21]. In a different study, the bacterium’s FT-IR spectrum was found to be identical to that of the SSC–12 EPS in (Figure 2), which also displayed the main polysaccharide peaks. The stretching of the –OH group is indicated by the broad band at 3379 cm^−1^, and the vibrations of C–H and sugar are at 2932 cm^−1^ and 1646 cm^−1^, respectively. The peaks at 1544, 1408, and 1026 cm^−1^ are indicative of the presence of amide, O–H, and C–O–C linkages, and the peak at 815 cm^−1^ is attributed to the presence of mannose [22]. The FTIR spectrum of EPS displays characteristic absorption bands reflecting its chemical composition, as mentioned in (Table 1). A broad signal around 3423.65 cm^−1^ indicates the presence of hydroxyl (–OH) groups, whereas peaks at 927.76 cm^−1^ and 815.89 cm^−1^ suggest glycosidic linkages and confirm the furanose form of the sugar residues. The lack of a noticeable peak between 1700 and 1770 cm^−1^ indicates that uronic acid residues are absent, and C–H stretching and bending vibrations were observed at 1230.58 cm^−1^ and 1406.11 cm^−1^, respectively. Furthermore, ^1^H NMR signals in the range of 3.1–4.4 ppm correspond to protons in the β-fructofuranoside structure with 2,6-linkages, corroborating the structural assignments suggested by the FTIR analysis [23]. On the other hand, high molecular weight and anionic nature (due to uronic acids or sulfates) of EPS influence viscosity, solubility, and emulsification ability. A marine microorganism, *Aspergillus* sp., produced an EPS (EPS-AG7) with a single, uniform molecular weight distribution as confirmed by SEC (Size Exclusion Chromatography) analysis. Its average molecular weight was estimated to be approximately 7.34 × 10^3^ Da, indicating a homogeneous polysaccharide fraction [24]. Similarly, marine *Streptomyces hirsutus* exhibited a high molecular weight, with mass average (Mw) and number average (Mn) molar masses of 4.25 × 10^5^ g/mol and 2.71 × 10^5^ g/mol, respectively, and a polydispersity index (PI) of 1.57. These values align with reported ranges (10–6000 kDa) [25].

## 3. Marine Microbiome

Microorganisms thrive in a wide range of natural environments [36]. They can grow in any biotope, even under harsh physical or chemical conditions. Examples of these environments are soil and the free water found in lakes and oceans [37]. By the close of 2016, researchers had cataloged nearly 28,500 distinct marine-derived natural compounds, encompassing a wide spectrum of chemical classes such as polysaccharides, peptides, polyketides, polyphenols, sterol-like molecules, and alkaloids, among others. Marine microorganisms developed unique adaptive mechanisms, including the development of potential defensive compounds, as a result of the severe environments that boosted genomic and metabolic diversity. Recent research has demonstrated that marine microbes can produce a wide range of distinct metabolites with diverse biological properties, anticipated to find application in the pharmaceutical, cosmetic, and medical sectors [38,39]. However, only a small number of culturable organisms have been identified and examined for their ability to produce extracellular polymeric substances, as mentioned in (Table 2). The biosynthesis of extracellular polysaccharides (EPSs) by marine bacteria is highly dependent on fermentation parameters. Furthermore, the composition of the medium, the availability of mineral salts, and the specific microbial strain, along with factors such as pH, temperature, oxygen levels, and the intensity of stirring, have a significant influence on the structure, chemical properties, and flow characteristics of the polymers produced [40,41].

Many marine microbes create EPS that may be used to fight bacteria and biofilms. For example, *Bacillus halotolerans*, a marine bacterium, makes EPS that is potent at stopping biofilm formation. It has a strong antibacterial action against *Pseudomonas* species, as shown by a significantly bigger inhibition zone than that of typical commercial antibiotics, and against *Serratia marcescens* and *Pseudomonas aeruginosa* clinical isolates [53]. *Pseudoalteromonas ulvae* is primarily located in marine settings and generates various inhibitory metabolic compounds, many of which remain unidentified. This bacterium was isolated for its ability to produce EPSs, which exhibit significant antibiofilm properties: the acidic fraction decreased biofilm formation by over 60% in all marine bacterial strains tested. The neutral fraction (PS I) was effective only against *P. mediterranea* TC7. However, the mixed fractions (TB-EPS) showed the highest level of inhibition, achieving up to 80% against *P. lipolytica* TC8 [54]. Red microalgae, especially those from the *Porphyridium* and *Rhodella* genera, have garnered interest because of their high levels of sulfated exopolysaccharides (EPSs). These polysaccharides have been found to possess significant biological activities, e.g., being able to inhibit the growth of bacteria, fungi, and biofilm formation with minimum inhibitory concentration (MIC) values of 62.5 to 1000 µg/mL. Additionally, at a concentration of 31.3 µg/mL, they can almost completely (90%) inhibit the formation of biofilm by *Candida albicans* [55]. Moreover, a marine *Enterobacter* strain was optimized to obtain a remarkable EPS yield of 8.6 g/L. The strain exhibited considerable antibacterial activity against *Staphylococcus aureus* and *Escherichia coli*, with the lowest concentration that could inhibit growth (MIC) of 15 mg/dL. On the contrary, it did not show any antifungal activity against Candida albicans and also did not present any prebiotic activity [56]. In addition, scientists found a sea bacterium, which is called *Vibrio* sp. BPM19, and is responsible for producing a galactan-like EPS. This compound is characterized by remarkable antimicrobial activity, which allows it to fight even drug-resistant types of *Salmonella typhi* and *Staphylococcus aureus* effectively [57]. On the contrary, the marine fungi have attracted the researchers’ interest due to their extraordinary capability to survive in very harsh environments and their talent to synthesize giant molecules of sugar (EPS) with remarkable biological activities. Among the different microorganisms, *Aspergillus terreus* was the one that not only produced the most EPS, with a yield of 4.98 g/L, but also exhibited the greatest potential in terms of bioactivity [58].

## 4. Molecular Mechanisms of EPS Regulation in Marine Microorganisms

An exopolysaccharide’s structural strength is mostly determined by the genetic makeup of the DNA in the microorganism [59]. The majority of bacterial species have a genetic locus located on a chromosome or plasmid where a specific gene or genetic cluster is principally responsible for producing extracellular polymeric substances (EPSs). There are a few bacterial regulatory genes that exhibit enhanced expression and EPS production, primarily through increased activity of enzymes like phosphoglucomutase, UDP-glucose pyrophosphorylase, and UDP-galactose 4-epimerase, which regulate nucleotide-sugar precursor synthesis [60]. EPS production is regulated at multiple levels in numerous marine bacteria. In the marine bacterium *Halomonas malpeensis*, at the metabolic level, the enzymes GalU, ManC, and Ugd regulate the production of precursors in response to sugar availability. At the genetic level, the regulatory proteins AlgA, MucR, and WspR recognize environmental stress and quorum sensing dynamically to regulate EPS-related genes. At the post-translational level, the three components of an ABC transporter (KpsM, KpsT, and KpsE) catalyze the action of inactivation based on ATP hydrolysis and feedback. In addition to the above, EPS production is also regulated environmentally, including factors such as salinity, type of nutrients, and organism stress [61]. Furthermore, numerous marine bacteria displayed distinct regulatory features controlling EPS biosynthesis. For instance, *Limnobacter alexandrii* LZ-4 primarily relies on the cellulose synthase operon (bcsA/B/C), showing strong activation under alkaline pH and sucrose or fructose supplementation. *Mesorhizobium alexandrii* LZ-8 contains the alginate pathway genes (algA/C/D/E), with elevated expression and EPS yield at higher temperatures. Strains *Nioella ostreopsis* Z7-4 and *Sulfitobacter alexandrii* AM1-D1 possess hybrid eps and wca operons, enabling production of mixed polysaccharides under variable carbon sources [62].

## 5. Mechanisms of Antibacterial and Antibiofilm Activity of Marine EPS

**Figure 3:**
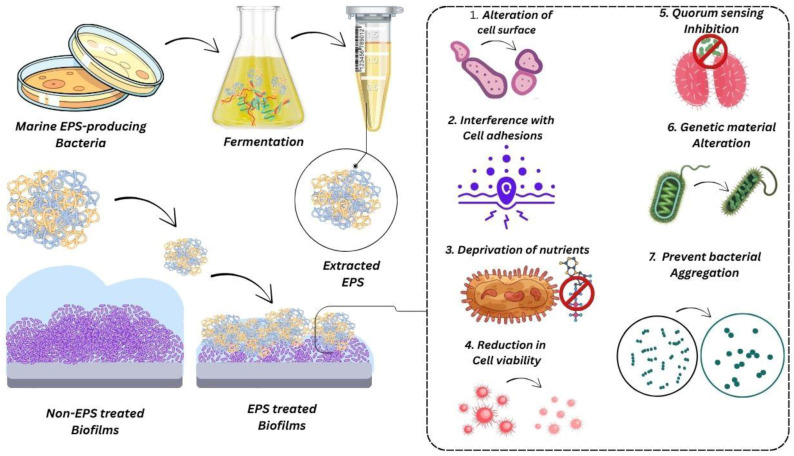
Proposed mechanism of marine bacterial EPS in biofilm inhibition. EPS extracted from marine bacteria disrupts biofilm formation by modifying cell surface properties, hindering adhesion, restricting nutrient access, impairing cell viability, suppressing quorum sensing, altering genetic response, and preventing bacterial aggregation.

Different EPSs have various physical and chemical attributes, thus providing a broad spectrum of product applications for the medical, agricultural, and personal care industries [63,64]. Exopolysaccharides (EPSs) can be classified into two main categories, namely homopolysaccharides and heteropolysaccharides [65]. The first ones, homopolysaccharides, are polymers of only one type of monosaccharide and can have linear or branched structures. Heteropolysaccharides, in contrast, are made up of two or more different sugar units. The characteristically different properties of EPSs are greatly determined by the sugar composition, molecular structure, and the mode of linkage between the sugar units. Consequently, the chemical structure of EPSs holds the key to their potential uses, such as in the case of preventing biofilm formation [66]. There are numerous pathways interpreted in various studies for the antibiofilm potential of marine EPS, as depicted in (Figure 3). These various materials could potentially reduce bacterial adhesion by making their surfaces less heterogeneous or by reducing the frequency of their contact, even though they do not directly support such an effect. The polysaccharides that are part of extracellular polymeric substances (EPSs) are thought to bring about changes in both non-living surfaces and the outer layers of bacteria, which may eventually determine the way biofilms are formed. One of the EPS types has also been reported to prevent the expression of bacterial genes that are important for biofilm formation [67]. On the contrary, there exists some important factors that contribute to their phenomenal ability to chelate. In other words, they have the capacity to bind and immobilize metals, trace elements, and nutrients, which finally results in the obstruction of microbial growth by starving such organisms of the resources required for their survival [68]. Moreover, EPS can influence cell-surface characteristics such as the charge and hydrophobicity of the surface. Furthermore, it plays the role of a dispersing agent, ensuring that cells do not adhere to each other or form clumps [69]. For instance, an exopolysaccharide isolated from marine *Bacillus licheniformis* B3-15 was recognized as a powerful inhibitor of adhesion and biofilm development of *K. pneumoniae*, *Str. pneumoniae*, *P. aeruginosa*, and *S. aureus*. The effect was noticeable with increased doses of exopolysaccharide. The phenomenon was caused by a bacterial surface modification followed by a reduction in the surface negative charges and a slight hydrophobicity change. These changes made bacteria less sticky and thus there was moderate inhibition of biofilm formation, especially in *K. pneumoniae* [70]. Similarly, *B. licheniformis* from the marine environment possesses several characteristics that make it possible to explore new anti-biofilm substances more effectively. The EPS produced by this bacterium showed antibiofilm activity linked to reduced cell viability or the production of quorum-sensing-like compounds [71].

## 6. Marine EPS: Synergy in Antimicrobial and Antibiofilm Activities

**Figure 4:**
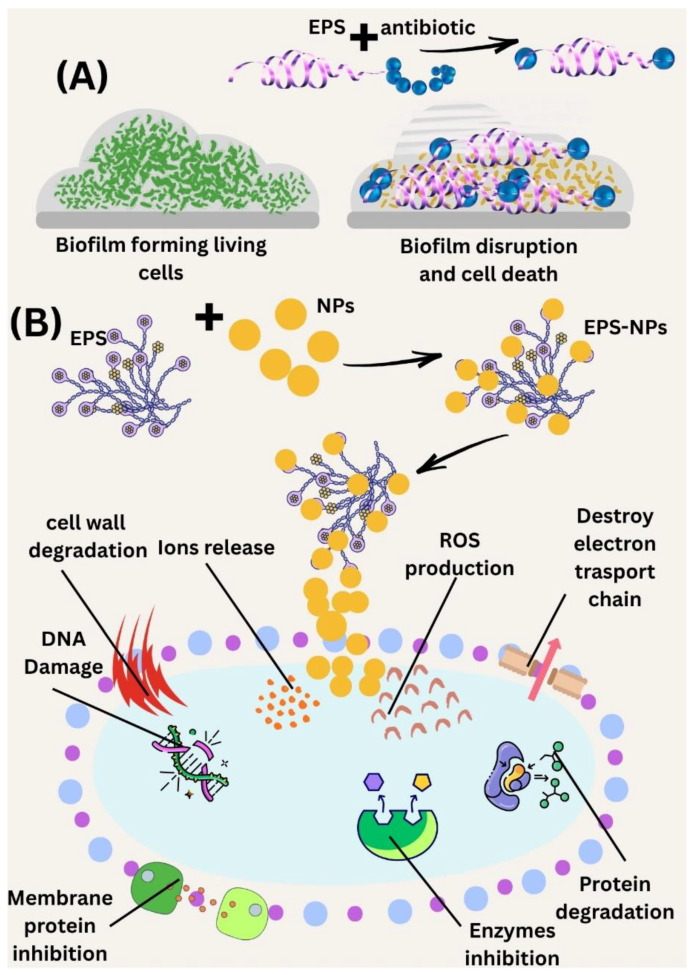
Schematic illustration of how biofilm inhibition works synergistically. (**A**) When antibiotics are combined with extracellular polymeric substances (EPSs), they disrupt the biofilm matrix, leading to the death of bacterial cells. (**B**) Nanoparticles team up with EPSs to create EPS–NP complexes, which trigger the production of reactive oxygen species (ROS), inhibit membranes and enzymes, break down proteins, and damage DNA. This all contributes to enhanced antimicrobial effects.

One significant factor influencing the formation of diverse biofilms is EPS, which causes either an inhibitory or synergistic effect on other microbial species. Numerous combined treatment approaches are using EPS with antibiotics and other materials synergistically, as depicted in (Figure 4). An *E. coli* EPS and tobramycin antibiotic were combined to target *P. aeruginosa* biofilms. Incorporating EPS notably enhanced the antibacterial effectiveness of tobramycin against these biofilms [72]. In contrast to antibiotics, a marine bacterium, *Bacillus licheniformis* B3-15, is used to extract exopolysaccharide (EPS B3-15), and biosurfactant (BS B3-15) demonstrated a clear synergistic effect in inhibiting bacterial attachment and dismantling established biofilms. The combined polymers effectively disrupted mature biofilms, exhibiting greater potency against *S. aureus* than *P. aeruginosa* [73]. The bacterium has exhibited an exceptional capacity to cooperate with vancomycin so as to eradicate MRSA biofilms. Their combination enabled the EPS to lower the MIC for vancomycin by four times and to realize a remarkable decrease of over 3 logs in the number of bacteria in just 24 h. Furthermore, modeling studies pointed out that the interaction of the components was very strong because of the ability of the EPS to dismantle the biofilm and to, at the same time, increase the penetration of the antibiotic, while also targeting different bacterial mechanisms to uplift its antibacterial potency [74]. At the moment, different types of metallic nanoparticles (NPs) are produced as capping agents with the help of microbial EPS. The microbial EPS not only facilitates the scaling up of production but also reduces the cost of manufacturing, increases the speed of the synthesis process, and improves the safety in making biocomposites. For example, *Rhodotorula mucilaginosa* EPS was used to create biocomposites that were found to be very efficient in getting rid of resistant strains of *Staphylococcus aureus* and *Pseudomonas aeruginosa* at the low concentrations of 3 mg/mL and 2 mg/mL, respectively. In addition, the biocomposite based on Zn-EPS was very effective in killing resistant *S. aureus* bacteria at a concentration of just 1 mg/mL. And most importantly, both biocomposites were non-toxic, thereby demonstrating their compatibility with living organisms [75]. In the same way, this bacterium is applied for the production of a biocomposite that merges EPS with nickel nanoparticles (Ni-EPS). It has been demonstrated that these materials can enhance the antimicrobial activity of different antibiotics with respect to multidrug-resistant strains of *S. aureus* and *P. aeruginosa*. Consequently, the findings reveal a powerful synergy as an eco-friendly nanomaterial in the battle against antibiotic resistance [76].

## 7. Novel Insights: Exploring Under-Researched Aspects

**Figure 5:**
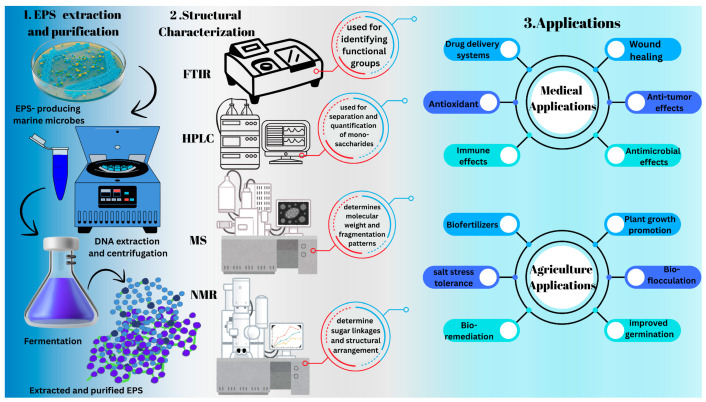
Schematic illustration of the step-by-step process of EPS isolation, extraction, purification, and structural characterization with potential applications in the biomedical and agricultural sectors.

EPS synthesis in both marine and terrestrial microbes has been significantly explored for various industrial-scale applications [77,78]. The ability of terrestrial microbes, for instance, lactic acid bacteria and PGPR, to produce EPS has been acknowledged as one of the principal factors responsible for the diversity of the structure and significant output from the use of inexpensive substrates. Nevertheless, the molecular mechanisms and genetic control of the synthesis of such terrestrial EPSs are still quite obscure as compared to marine EPS, thus impeding their optimization [79]. In addition, EPS synthesized by terrestrial (non-marine) bacteria are primarily valued for their rheological and functional characteristics, whereas those obtained from marine bacteria are predominantly exploited for their diverse biological activities [80]. Marine EPSs are vital, safe, natural biopolymers that are utilized in a variety of applications, as depicted in (Figure 5). However, more work is needed to produce these EPSs on an industrial scale [81]. The research in this area remains limited, particularly for EPSs synthesized by microbes inhabiting extreme marine environments. Furthermore, A wide range of marine microorganisms, including *Vibrio alginolyticus*, *Bacillus* spp., *Microbacterium aurantiacum*, *Rhodobacter johrii*, *Pseudoalteromonas* sp., *Alteromonas* sp., *Neorhizobium urealyticum*, *Natronotalea sambharensis*, and marine-derived fungi such as *Aspergillus* and *Penicillium* species, have been reported to produce structurally diverse exopolysaccharides (EPSs). Notably, many of these marine microbes—such as *Natronotalea sambharensis*, *Microbacterium aurantiacum*, and *Neorhizobium urealyticum*—are underexplored, particularly in the context of antibiofilm and antibacterial activity [82,83]. During the past few years, marine organisms have attracted the interest of researchers because they are the expected and main sources of bioactive substances for health-related applications. Solvents of various marine species being isolated have exhibited the strength of huge therapeutic effects, which include immunomodulatory, antibacterial, and antibiofilm effects [84]. Marine EPSs show, through their amazing immunomodulatory properties, that such substances can indirectly help in the control of biofilm formation, e.g., through cytokine expression regulation, boosting host defense reactions, and altering the molecular recognition pathways that are involved in the adhesion and colonization of microbes [85].

## 8. AI-Driven Insights into Marine Exopolysaccharides

As marine scientific study advances into a new age of intelligence and continuously enhances marine data, artificial intelligence (AI) can proficiently harness the latent knowledge embedded in extensive datasets. Consequently, it is increasingly attracting the interest of marine life scientists [86]. Recently, AI was used as an advanced modeling and optimization tool to identify how different medium components influence EPSs production from *Bacillus licheniformis* isolated from a hot water spring. For this purpose, a hybrid support vector machine (h-SVM) model was developed to capture complex variable interactions more accurately than traditional RSM. This AI-driven strategy led to a ~5-fold increase in EPS yield [87]. Another AI, an artificial neural network (ANN), was used to predict and optimize EPS production in Bacillus spp., learning the nonlinear relationships between medium components and EPS yield, especially when biomass was included as an input variable. The optimized ANN model (EPS-NN2) showed very high predictive accuracy (R^2^ = 0.98) with relatively low error [88]. The integration of ANN AI-based, powerful technology with statistical optimization provided a more powerful strategy for maximizing EPS production from *Leuconostoc mesenteroides* ABNFT-1. Its predictions surpassed the RSM-optimized yield, demonstrating higher accuracy and better modeling of nonlinear interactions [89]. Furthermore, AI was used through a genetic algorithm-optimized artificial neural network (GA-ANN) to model and improve EPS production in *Haloferax mediterranei*. The ANN learned complex nonlinear effects of sucrose, yeast extract, and urea on both biomass and EPS yield, outperforming classical regression and RSM [90]. This AI modeling identifies the complicated association between the parts of the medium and the EPS yield that conventional methods fail to recognize. After being trained, it suggests the best culture conditions for the increase in EPS production. Its complete reliance on data makes it a good tool for fermentation optimization, as it can work with difficult biological systems [91].

## 9. Potential Application of Marine EPS in Biomedicine

Among the large macromolecules that are being studied a lot is microbial extracellular polysaccharide (EPS), which is one of the most important substances for the biomedicine and pharmaceutical industries. In the course of the past two decades, the chemical composition of unique marine exopolysaccharides (EPSs) has determined their functionality and thus, opened up a wide range of potential uses in industrial, medical, and environmental sectors [40], which are given below.

### 9.1. Wound Healing Potential as Coatings

**Figure 6:**
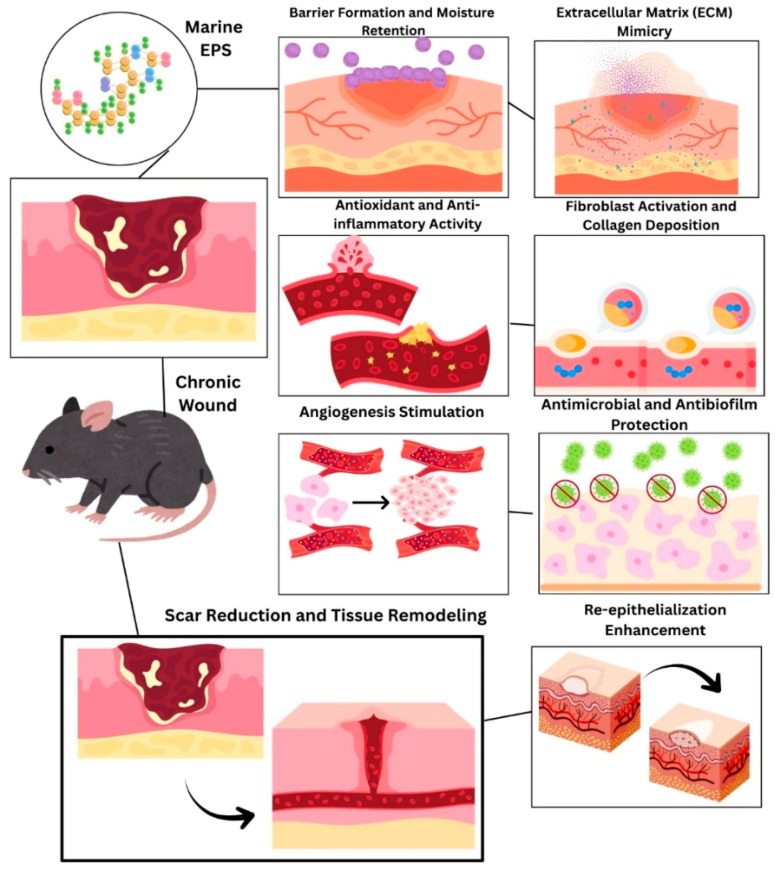
Schematic illustration of the wound-healing mechanism promoted by marine-derived exopolysaccharide (EPS) coating. The EPS film forms a protective, moisture-retaining layer that mimics the extracellular matrix, enhances fibroblast proliferation, collagen synthesis, and angiogenesis, and supports re-epithelialization. Its intrinsic antioxidant, anti-inflammatory, antimicrobial, and antibiofilm properties accelerate tissue regeneration and minimize scar formation, ensuring rapid and infection-free healing.

Marine-derived exopolysaccharides (EPSs) have some excellent properties, such as high absorption ability, good biocompatibility, and low cost, which make them the most favorable materials for use in wound dressings and tissue engineering. Natural EPS, unlike synthetic polymers, is the best at mimicking the extracellular matrix, thus allowing the access of tissues to the metabolism, improving cellular activities such as proliferation, migration, and differentiation during tissue repair, as depicted in (Figure 6). It was a marine EPS ointment/coating made through *Enterococcus faecium* that showed the strongest healing power of wounds with about 94.93% closure on the seventh day and total healing (100%) on the twelfth day [92]. Likewise, the EPS coating produced by the marine microorganism *Halomonas malpeensis*, a member of the group of microorganisms living in extreme environments, demonstrated excellent efficacy in the healing of burn wounds. Moreover, an incredibly high wound contraction percent of 80.6 ± 9.4% was achieved, which is greater than the positive control’s 54.6 ± 8.0% and the untreated group’s 49.2 ± 3.7%. In addition, the histopathological examination confirmed that significant growth of epidermal tissue occurred after just 15 days of treatment [93]. Another marine microorganism, *Polaribacter*, has proven to stimulate fibroblasts’ migration, speed up the healing process of wounds, and also assist in tissue regeneration in Sprague–Dawley rats. In addition, it was a major factor in the healing of frostbite wounds and strengthened the skin to resist the adverse effects of the cold [94]. As far as the antimicrobial activities are concerned, EPS-Ca6 from *Lactobacillus* sp. Ca6 revealed strong antioxidant and antibacterial activities, thereby blocking the growth of not only *Salmonella enterica* but also *Micrococcus luteus*. During the trials with rats having excision wounds, it became evident that EPS-Ca6 helped the healing process remarkably, and the animal was fully restored in a mere 14 days. Histological assessments verified that the process of re-epithelialization was total and the epidermis was completely regenerated [95]. A different type of oceanic bacterium, *Bacillus rugosus*, is the one that has been remarkable in terms of wound healing since it has been able to give cellular migration a boost and heal the wound to an astounding 72.66% in just 48 h. This bacterium’s antibacterial properties are so powerful that they can easily fight off both Gram-positive and Gram-negative pathogens. In addition to that, it also has a very high 95.6% inhibition rate against *Helicobacter pylori* in terms of biofilm formation prevention. All these astonishing characteristics point to the fact that EPSR9 not only promotes tissue regeneration but also takes care of bacterial infections and complications from biofilms that come up during the healing process [96].

### 9.2. Marine EPS as Drug Delivery Systems

Marine substances that have unique characteristics are utilized in different industries, such as pharmaceuticals and biomedical research. Biomaterials made from these substances are exceptionally compatible with living beings, naturally decomposable, and safe for human application. Their natural occurrence makes them a perfect choice for drug delivery systems that facilitate the movement of bioactive molecules [97]. *Trichormus variabilis*, a marine microorganism, has significant potential as a system for delivering drugs and biomolecules. Researchers developed a photopolymerizable hybrid hydrogel (REPS-Hy) by combining polyethylene glycol diacrylate (PEGDa) with extracellular polysaccharides (EPSs) extracted from *Trichormus variabilis*. These hydrogels from marine EPS were created with the enzyme thiosulfate: cyanide sulfur transferase (TST) and shown to have continuous enzymatic activity, thus being suitable for enzyme delivery. Moreover, the ingredients of the hydrogel were a factor in the binding of human mesenchymal stem cells (hMSCs) and an increase in their viability and growth through cell adhesion and survival [98]. *Cyanothece* is a sea microbe with the potential to be a natural carrier for medicinal biomolecules. The EPS produced by it has demonstrated its capability to self-assemble and create a strong structure around the proteins, thereby shielding the therapeutic compounds from being destroyed. One of the most important features of this structure is that the proteins do not lose their activity during the process of release, which is accomplished by swelling under physiological conditions that result in a slow and mild delivery. Changing the release rate can be achieved by adding divalent cations like calcium, which gives the timing of the release very accurate control [99]. Moreover, EPSs made from marine exopolysaccharides (GY785 EPS) were highlighted to be an efficient means of transporting TGF-β1 in cartilage repair because of their accurate and controlled release properties. The combination of TGF-β1 with sulphated EPS derivatives through encapsulation or complexation not only results in a controlled release but also preserves the growth factor’s biological activity, thus making these biopolymeric systems suitable for next-generation tissue engineering applications [100].

### 9.3. Marine EPS Potential in Agriculture

**Figure 7:**
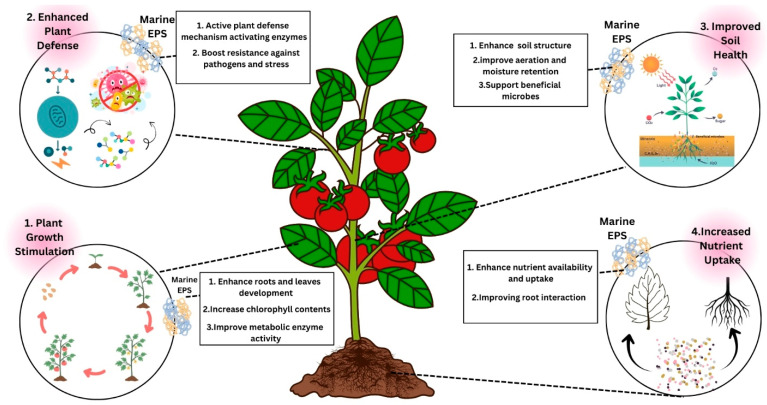
Schematic representation of the beneficial effects of marine-derived exopolysaccharides (EPSs) on plant growth and soil health. Marine EPS enhances root and leaf development, chlorophyll content, and metabolic enzyme activity, stimulating overall plant growth. They activate plant defense mechanisms, strengthening resistance to pathogens and stress. EPS also improves soil structure, aeration, and water retention while promoting beneficial microbial activity, resulting in improved soil fertility and enhanced nutrient uptake efficiency.

Microorganisms are the primary producers of EPS in the major agricultural domain, which consequently leads to creating good environmental and soil conditions by means of different chemical reactions, nutrient storage, and protection against drought and salinity. The microorganisms, by means of their clumping, develop an advantageous soil structure while at the same time forming a protective environment allowing for the availability of moisture and nutrients as depicted in (Figure 7) in sufficient amounts for plant growth [101]. *Arthrospira platensis*, an oceanic microbe, produces an EPS that promotes the development of tomato plants. The use of this microorganism positively influenced the morphological parameters evaluated in this study and was also responsible for the generation of better quality and larger amounts of nutrients and pigments in plants (increased fruit protein and carbohydrate content). Furthermore, the phosphorus level in the plants increased, indicating that nutrient uptake and transport processes were enhanced. The application of polysaccharides on soil surfaces showed that this method had superior plant growth promotion capability as compared to other treatments [102]. *Gloeothece verrucosa*, another marine microbe, released exopolymeric substances (EPSs) that have been used on plants like *Arabidopsis thaliana* and tomatoes, which activate defense responses by raising phenylalanine ammonia-lyase (PAL) activity, and by proline and anthocyanins. Overall, EPS from *G. verrucosa* functions as a bio-stimulator that promotes plant growing, stress tolerance, and the surrounding soil environment health [103]. The red microalga *Porphyridium sordidum* has been found to produce marine EPS that holds great potential as a natural material for the activation of plant defense systems in sustainable agriculture. The polysaccharides recognized as bioactive may incite the activation of plant resistance signaling pathways, making use of salicylic acid and later on developing plant immunity. The polysaccharide application of the plant can be considered a way to protect them from several pathogens, like *Fusarium oxysporum*, while simultaneously cutting down the use of chemical pesticides. The role of EPS in agriculture is through the enhancement of soil and health of the plant by the stimulation of beneficial microorganisms and the improvement of nutrient absorption [104]. Natural biostimulants in the form of Exopolysaccharides (EPSs) originating from *Dunaliella salina* increase the capability of tomato plants (*Solanum lycopersicum*) to cope with salt stress. The sulfated EPS from *Dunaliella salina* improved the situation of the plant during the salt stress period by plant growth enhancement, potassium levels maintenance, and oxidative damage protection through enzyme modulation and osmoprotectant activity exploitation [105].

## 10. Impact of Climate Change on Marine EPS-Producing Microbiota

**Figure 8:**
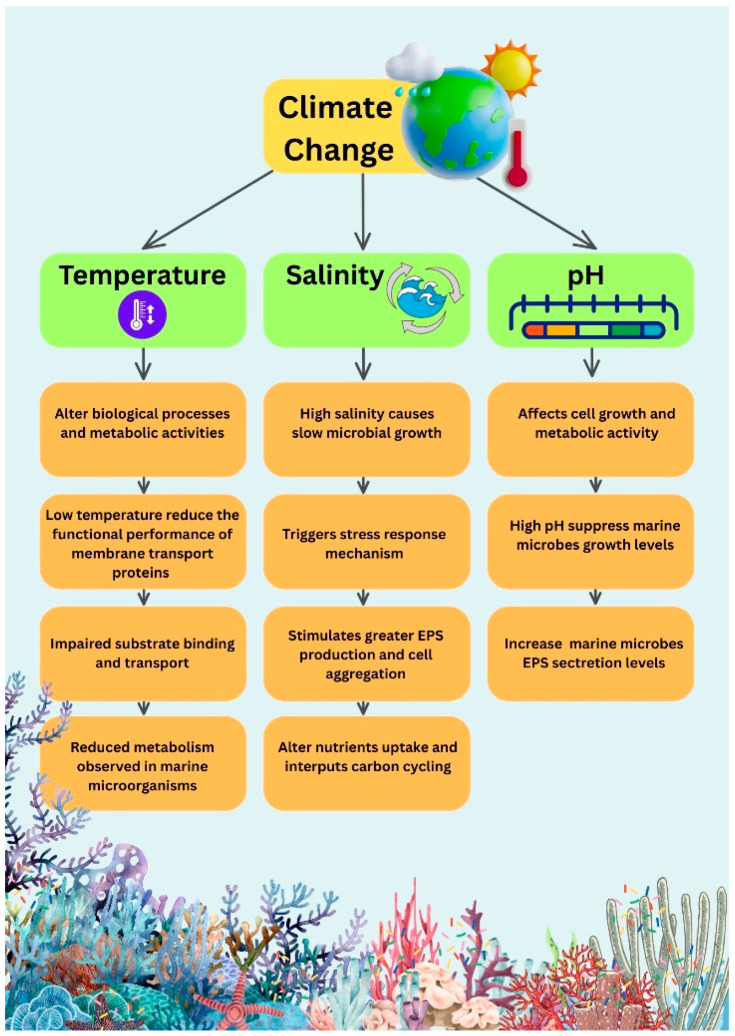
Schematic illustration of climate-change-driven environmental stressors and their impacts on microbial extracellular polymeric substances (EPSs) regulation in marine ecosystems. Variations in temperature, salinity, and pH alter microbial physiological processes, including growth rates, metabolic activity, membrane transport, nutrient uptake, and stress response pathways. These physiological disruptions collectively modify EPS production dynamics, influencing cell aggregation and carbon cycling within marine environments.

It is important to understand that marine microbes frequently experience the same problems as animals, plants, and other species due to changes in the climate. The yield of EPS is impacted by the effects of climate change, including rising temperatures, extreme weather, and rising levels of carbon dioxide [106]. Furthermore, the components of carbon and nitrogen that microbes used were linked to the release of EPS. The external surroundings, including salt content, pH, and temperature, also had an impact, as illustrated in (Figure 8). In addition to having a significant impact on these microbes’ metabolic processes, temperature fluctuations are one of the most significant environmental elements that have an impact on the biological characteristics of bacteria. When the temperature drops too low, the functional performance of membrane transport proteins declines, reducing their ability to bind and transport substrates effectively. Therefore, the reduced temperature leads to inhibition of the overall metabolic rate and EPS production, which were found for the marine bacterium *Bacillus* sp. ZT-1 [107]. In the same manner, the marine microorganism *A. platensis*’s EPS production was similarly influenced by pH. Thus, the highly alkaline condition (pH 11.5) not only inhibited the growth of cells but also, very surprisingly, increased the amount of EPS secretion. [108]. In addition, one of the factors that affects EPS production in marine microbiota, for example, in *Synechococcus*, is the high salinity exposure. The presence of high salts not only hindered the growth of the cells but also led to the production of more EPS and cell clustering, which is a protective response and at the same time has an impact on the food webs and carbon cycling in the oceans [109]. Consequently, the chief causes behind the changes in ambient conditions and light, such as rising temperatures, fluctuating light intensity, altered nutrient availability, and CO_2_-absorbing ocean, played a decisive role in the marine microbe’s photosynthetic efficiency, biomass production, and metabolic activity. Eventually, that change in the conditions of the microorganisms’ environment leads to a change in their biochemical pathways and thereby the whole ecosystem’s productivity [110].

## 11. Conclusions and Future Perspectives

The marine microbiome, unexplored EPSs with unique functions and therapeutic potential, has amended the research interests. The marine microbial community produces an abundance of EPS forms with numerous bio-activities, i.e., antimicrobial properties and modulatory effects on the host immune system. Marine microorganisms display antioxidant and anti-inflammatory properties. Biomedical applications for these polymers have unarguably been made possible by their use in wound healing, drug delivery, and tissue regeneration. Marine EPSs have diverse physicochemical properties which distinguish them from classical polysaccharides of terrestrial origins and make them attractive candidates for the construction of advanced drug delivery systems due to their potential in hydrogel formation and controlled release of different biomolecules. Marine EPSs have promising applications to improve the water- and nutrient-retention of soil and to increase the plants’ tolerance to stress, although their applications in these fields are still under-explored and their potential has yet to be fully realized. Although marine bacteria harbor rich EPS biosynthetic potential, genome-guided strategies to enhance EPS yield remain scarcely explored. Current research primarily focuses on strain isolation and process optimization, while genetic engineering and AI-driven predictive approaches are still in their infancy. Limited biomass productivity, cryptic biosynthetic gene clusters, and challenges in large-scale cultivation further restrict progress. Therefore, integrating advanced genomics with artificial intelligence offers a promising yet underexplored approach to boosting marine EPS production.

Future research directions should also include the development of large-scale production methods, elucidation of the molecular mechanisms involved in biosynthesis, and clinical studies to assess the therapeutic effects of marine EPS. The combination of marine EPS with nanoparticles and small molecules has the potential to provide new hybrid materials for drug delivery and antimicrobial applications. Moreover, variations in climate factors and molecular regulation significantly influence EPS biosynthesis and biochemical composition, shaping their structural integrity and antibacterial potential under an evolving marine ecosystem.

## Figures and Tables

**Table 1:** Structural and functional characteristics of selected marine microbial EPSs.

EPS Name	Source Organism	Linkage Type	Monomer Composition	Key IR Signatures (cm^−1^)	Functional Properties	References
EPS B3-15	*Bacillus licheniformis* B3-15	α-(1→4) glycosidic linkages	Repeating disaccharide units with manno-pyranosidic configuration and low protein content; includes poly-γ-glutamic acid (γ-PGA)	Major EPS peaks: 3500–3200 (O–H/amide A), 1647 (C=O), 1542 (N–O from γ-PGA), 1200–950 (exopolysaccharide region), 872.7 (glycosidic bond)	Heavy metal adsorption	[26]
EPS5SH	*Bacillus* sp. H5	α-(1→4)-Manp, α-(1→2)-Manp, α-(1→4,6)-Manp, β-terminal-Manp	Mannose 1.00, Glucosamine 0.02, Glucose 0.07, Galactose 0.02	Major EPS peaks around 3400 (O–H), 2920 (C–H), 1640 (C=O), 1050–1150 (C–O–C, glycosidic)	Immunostimulatory mechanism activation	[27]
EPS	*Saccharophagus degradans*	Not specified	Glucose, Mannose, Galactose	Major EPS peaks around 1000–1200 (C–O, carbohydrates), 1637 (C=O, carboxyl), 1550 (amide II, protein), 1742/1262 (O-acetyl esters)	Carbon source and C/N ratio-dependent production	[28]
RSK CAS4 EPS	*Bacillus thuringiensis* RSK CAS4	Not specified	Fructose, Galactose	–OH (3433 cm^−1^), C–H (2954, 2926 cm^−1^), C=O (1633 cm^−1^), C–O–C glycosidic linkages (1122 cm^−1^), and C–H bending (615 cm^−1^)	Antioxidant, antitumor activities	[29]
EPS	*Enterobacter cloacae* marine isolate B8	glycosidic linkages (C–O–C)	Mainly glucose with trace mannose (Glc: Man ≈ 1:0.015)	Not specified	Antioxidant activity	[30]
EPS	*Streptomyces carpaticus*	C–O–C	Galactouronic acid, Glucose, Xylose, Galactose, Mannose, Fructose = 3:1:1:2:2:1	Major peaks around 1380 (–COO–, sulfate ester), 1353 (monosaccharide sulfates), 836 (α-glycosidic bonds)—indicating an α-type acidic heteropolysaccharide	Anticancer-activity	[31]
K1^T^-9	*Neorhizobium urealyticum*	Heteropolysaccharide	Galacturonic acid, Glucose	3399 (OH), 2933.8 (C–H), 2099.8 (C≡C), 1644.3 (COO^−^), 1408 (C–O–H), 1200–1000 (glycosidic bonds/pyranoid ring), 617.2 (fingerprint)	Antioxidant activities	[32]
EPS-B3-15	*Bacillus licheniformis* B3-15	α-(1→4) glycosidic linkages	Mannose, Glucose	glycosidic linkages, pyranosidic monosaccharides, OH stretching (3500–3000 cm^−1^), C=O at 1665 cm^−1^, and CH at 2061 cm^−1^	Thermostable up to 78.5 °C	[33]
EPS-BFB-6S	*Pseudomonas sihuiensis* BFB-6S	α/β-glycosidic	Mannose, Glucose, Fructose	Amide: C–N, C–C, C=O, –NH bending; polysaccharide peaks dominant	Metabolic modulation under high pCO_2_, Stress adaptation	[34]
EPS-CDR-SL 7Cii	*Rhodobacter johrii*	1,6 α-D-Glcp, 1,4 β-D-Glcp, 1,3 β-D-GlcA, 1,3 β-D-Galp, 1,6 β-D-Galf, 3 α-L-Rhmp	Glucose: Glucuronic acid: Rhamnose: Galactose = 3:1.5:0.25:0.25	Major Peaks at 3398 cm^−1^—OH (hydroxyl), 1735 cm^−1^—diacyl ester/glucuronic acid, 1608 and 1414 cm^−1^—COO^−^ (carboxyl), 1041 cm^−1^—C–O–C glycosidic bonds	Bio-emulsifier applications	[35]

**Table 2:** Marine microorganisms reported for exopolysaccharide (EPS) production, including their synthesis approaches, yield, and key biological or functional outcomes.

Marine Microorganism	Production Method	Key Factors	Results/Properties	EPS Yield	References
*Streptomyces vinaceusdrappus* strain AMG31	Submerged fermentation	Acidic EPS, High uronic acid content: 39.77%, High sulfate content: 18.8%, Monosaccharide ratio: Arabinose: Glucose: Galacturonic acid = 0.5: 2:2	Antioxidant, Anti-inflammatory, and Enzyme-inhibitory activity	10.6 g/L	[42]
*Actinobacterium Streptomyces violaceus* MM72	submerged fermentation	Molecular weight of 8.96 × 10^5^ Da	Strong antioxidant activity, including DPPH radical scavenging, superoxide scavenging, and metal chelation. Exhibited moderate lipid peroxidation inhibition and reducing power, suggesting potential for natural antioxidant applications	Not specified	[43]
*Bacillus subtilis* strain AG4,	fractionation and precipitation processes	Sulphated β-glycosidic heteropolysaccharide; molecular weight 1.48 × 10^4^ g/mol; monosaccharide composition: glucose, rhamnose, and arabinose (5:1:3)	High crystallinity and porosity, strong antioxidant activity, cytotoxicity against multiple cancer cell lines, anti-inflammatory effects (LOX, COX-2, membrane stabilization), and acetylcholinesterase inhibition	8.12 g/L	[44]
*Bacillus cereus*	Not specified	Monosaccharide composition: glucose, galacturonic acid, and arabinose (2.0:0.8:1.0), uronic acid content 28.7%, no sulfate	Strong antioxidant activity (DPPH IC_50_ ≈ 500 µg/mL; H_2_O_2_ IC_50_ ≈ 1500 µg/mL); cytotoxicity against T-24, MCF-7, and PC-3 cancer cells; potent anti-inflammatory activity (LOX IC_50_ ≈ 12.9 µg/mL; COX-2 IC_50_ ≈ 29.6 µg/mL); antibacterial activity against MRSA and coagulase-negative *staphylococci*	7.95 g/L	[45]
*Bacillus velezensis* AG6	Not specified	Monosaccharide composition: xylose, galactose, and galacturonic acid in a molar ratio of 2:0.5:2	Strong antioxidant activity in DPPH, H_2_O_2_, and ABTS assays, showing dose- and time-dependent inhibition (91.3%, 80.2%, and 75.3% at 1500 μg/mL), inhibition of six cancer cell lines, anti-inflammatory effects via LOX and COX-2 inhibition; antimicrobial and antibiofilm activities	5.79 g/L	[46]
*Pseudomonas* sp. *strain* AHG22	Cultured in broth solution	Potent DPPH-scavenging profile indicated by an IC_50_ of 46.99 μg/mL	Moderate anti-inflammatory activity through 5-LOX and COX-2 inhibition; antidiabetic, anti-obesity, neuroprotective, antibiofilm, and broad-spectrum antibacterial properties with MBC/MIC ≤ 2	6.98 g/L	[47]
*Kocuria* sp. *strain* AG5	Cultivation/fermentation	Molecular weight of approximately 4.9 × 10^4^ g/mol; composed primarily of glucose, galacturonic acid, arabinose, and xylose	Potent antioxidant activity reaching 98% at 2000 µg/mL, notable anti-inflammatory effects with 5-LOX IC_50_ = 15.39 µg/mL and COX-2 IC_50_ = 28.06 µg/mL, cytotoxicity against cancer cell lines, hemolysis suppression, moderate acetylcholine esterase inhibition	6.84 g/L	[48]
*Bacillus subtilis strain* SH1	one-factor-at-a-time experiments and Response Surface Methodology (RSM)	Multifunctional bioactivity under different concentrations to determine dose-dependent effects	Antibacterial activity against *S. faecalis*, significant antitumor effects on MCF-7, HCT-116, and HepG2 cells, antiviral activity at 500 µg/mL	33.8 g/L	[49]
*Bacillus amyloliquefaciens* 3MS 2017	Not Specified	Explored multifunctional bioactivity, including antioxidant mechanisms (ROS scavenging, metal chelation), selective COX inhibition, and anticancer effects. In vitro studies were conducted on MCF7, PC3, and EAC cells, and in vivo evaluation in EAC-bearing models (oral, 200 mg/kg)	Exhibited strong antioxidant activity via ROS scavenging and metal chelation, weak reducing power, selectively inhibited COX-2, showed high in vitro anticancer activity against MCF7 (65.2% death; IC_50_ = 70 μg/mL), reduced PC3 and EAC cell viability, and significantly slowed EAC progression in vivo	Not Specified	[50]
*Bacillus subtilis* MKU SERB2	Response Surface Methodology (RSM)	11.5 g/L sucrose, 3.5 g/L yeast extract, 3.0 g/L peptone, 2.5 g/L CaCl_2_. No detectable hemolytic or lymphocyte toxicity, indicating safe biomedical applicability	EPS exhibited strong antioxidant activity, moderate anticoagulant potential	617.81 μg/mL	[51]
*Lactiplantibacillus plantarum* C7	MRS broth cultivation	No molecular weight specified, Prebiotic score 0.043, Carbohydrate content 3.679 g eq glucose/L	Antioxidant, antibacterial, and enzyme inhibition activities	0.2 to 0.9 g/L	[52]

## Data Availability

No new data were created or analyzed in this study. Data sharing is not applicable to this study.

## Abbreviations

The following abbreviations are used in this manuscript:

EPSs	Exopolysaccharides
AMR	Antimicrobial resistance
NPs	Nanoparticles
SEC	Size Exclusion Chromatography
MIC	Minimum inhibitory concentration
